# Influence of Purple Onion Pulp Addition Level on Oxidative, Microbial, and Sensory Characteristics of Refrigerated Beef Patties

**DOI:** 10.3390/foods14213659

**Published:** 2025-10-27

**Authors:** Jiaxin Wei, Fujuan Zhang, Li Yang, Cuntang Wang

**Affiliations:** 1 College of Food and Bioengineering, Qiqihar University, Qiqihar 161006, China; 2 Engineering Research Center of Plant Food Processing Technology, Ministry of Education, Qiqihar 161006, China; 3 College of Biological Food Engineering, Chaoyang Normal University, Chaoyang 122000, China; perfect283@sohu.com

**Keywords:** onion pulp, prepared BP, metmyoglobin, lipid oxidation, protein oxidation

## Abstract

Pre-treated beef patties (BPs) face storage instability due to lipid/protein oxidation, microbial spoilage, and quality loss. Purple onion pulp (OP), a bioactive-rich product, offers potential as a natural preservative. This study evaluated the utilization of OP (2.5%, 5.0%, 10.0%) in BPs for refrigerated (4 °C) stability. The results showed that during storage, OP addition reduced the pH value, a* value, and b* value of beef, while cooking loss was not significantly affected. At the end of storage, the addition of 10.0% OP decreased the formation of metmyoglobin (MetMb), thiobarbituric acid reactive substances (TBARSs) (with a reduction rate of 15.96%), and carbonyl groups, and also inhibited spoilage bacteria. Sensory evaluation and texture analysis indicated that the addition of OP improved the hardness, juiciness, and odor of beef. Specifically, the incorporation of 10.0% OP extended the shelf life of BPs to 9 days, effectively improving their storage stability. Therefore, adding 10% purple onion pulp OP to BPs can improve the storage stability and sensory quality of refrigerated BPs.

## 1. Introduction

Beef patties (BPs) constitute a staple ready-to-eat meat product, serving as an essential component within fast-food industries, home kitchens, and food service supply chains. Their widespread adoption is largely attributed to their high nutritional density and versatility in cooking applications [1,2]. However, refrigerated storage presents significant challenges, primarily due to quality deterioration driven by lipid and protein oxidation [3]. Harmful products generated by lipid oxidation in meat, including aldehydes, ketones, and alcohols, impair the product’s flavor. Meanwhile, protein oxidation leads to meat discoloration (attributed to myoglobin oxidation) and texture deterioration (caused by myofibrillar protein oxidation) [4]. Crucially, compared to whole muscle meats, minced BPs exhibit heightened susceptibility to intense oxidation. The mincing process incorporates substantial oxygen, accelerating detrimental processes such as meat browning, texture degradation, flavor impairment, and nutrient loss [5]. Concurrently, protein oxidation induces conformational and functional alterations in myofibrillar proteins, leading to phenomena like protein cross-linking or proteolytic degradation. These changes adversely affect key quality attributes, including texture, water-holding capacity, and nutritional integrity [6]. Compounding the issue, research indicates that lipid and protein oxidation in BPs escalate markedly within just 1–2 weeks under standard refrigeration (4 °C), severely curtailing shelf life [7]. Consequently, developing effective strategies to inhibit these oxidation reactions during refrigerated storage has become paramount to preserving quality and extending shelf life.

In response to these challenges and driven by consumers’ heightened awareness of food safety and preference for natural products, the food industry has increasingly sought natural antioxidants as alternatives to synthetic counterparts [8]. Although synthetic antioxidants, such as butylhydroxyanisole (BHA) and dibutylhydroxytoluene (BHT), demonstrate potent efficacy, concerns regarding their long-term safety have led to usage restrictions in some regions [7]. In contrast to their synthetic counterparts, natural antioxidants—particularly plant-derived extracts—offer advantages such as wide availability, high safety, and additional health benefits (e.g., anti-inflammatory and antibacterial) [9]. This is largely due to their richness in bioactive compounds like phenolics and flavonoids, which make them highly promising for preserving meat products [10,11].

Plant polyphenols, a major class of secondary metabolites abundant in various plant parts (e.g., skin, roots, leaves, fruits) [12], have a variety of biological functions. These encompass potent antioxidant, antibacterial, anticancer, and anti-inflammatory effects, alongside roles in regulating blood sugar, lipids, and body weight [13]. The core mechanism underlying their antioxidant efficacy lies in the phenolic hydroxyl group (-OH), which effectively scavenges free radicals by converting them into stable compounds, thereby terminating chain reactions and mitigating oxidative damage [13]. Specifically within meat systems, plant polyphenols can effectively inhibit lipid and protein oxidation, reduce fatty acid spoilage, delay protein degradation, and suppress microbial growth, collectively contributing to enhanced quality preservation and extended shelf life of meat products [10,11]. Numerous studies substantiate this potential, demonstrating that incorporating antioxidant-rich fruits and vegetables—such as red pepper [14], Agaricus bisporus mushroom [15], Opuntia ficus-indica fruits [16], and Açaí residue [17]—effectively combats lipid and protein oxidation in meat matrices.

Onion (*Allium cepa* L.), a ubiquitous culinary ingredient valued both as a condiment and a rich source of bioactive components (e.g., quercetin, catechin, flavonoids, organic sulfur compounds), possesses notable antioxidant, anti-inflammatory, and antibacterial properties [18,19]. Significantly, among onion varieties, purple onions exhibit enhanced antioxidant activity, attributable to their higher anthocyanin content [20,21]. Given these properties, the application of onion extracts in food preservation has garnered increasing interest. Onion extracts effectively inhibited lipid oxidation (measured by reduced TBARS values) in emulsion sausage [22], while onion powder improved antioxidant properties in fish sausages [23]. However, despite this emerging evidence, the utilization of onion, particularly OP in BPs, remains relatively unexplored. Crucially, a systematic investigation into the influence of varying OP concentrations on the lipid/protein oxidation and sensory characteristics of refrigerated BPs represents a significant research gap.

A critical factor influencing the efficacy of natural antioxidants like plant polyphenols is their dosage [11,12]. Suboptimal concentrations may yield insufficient antioxidative effects, while excessive addition can induce undesirable off-flavors and discoloration, ultimately compromising sensory acceptability [7,12]. Therefore, this study aims to systematically investigate the dose-dependent effects of purple OP supplementation on lipid/protein oxidation dynamics and sensory attributes of refrigerated BPs. Specifically, through quantitative tracking of key oxidation markers—thiobarbituric acid reactive substances (TBARSs), peroxide value (PV), and protein oxidation (carbonyl content, sulfhydryl group concentration)—alongside standardized sensory evaluation during cold storage, we seek to determine the optimal inclusion level. The findings are expected to provide a scientific basis for utilizing purple onion pulp as a natural antioxidant in beef product formulations and offer innovative technical solutions for developing high-quality, naturally preserved meat products. Defining this critical threshold that optimally balances antioxidative efficiency with sensory integrity constitutes the core objective, holding significant theoretical and practical implications for meat science.

## 2. Materials and Methods

### 2.1. Materials

The fresh beef *longissimus dorsi* muscle at 24 h post-slaughter (pH 5.8) and peeled onions (purple onions of the same variety harvested one month later) were purchased from a local market in Qiqihar, China. All samples were transported to the laboratory in a cold bag at low temperature.

2-Thiobarbituric acid (2-TBA), Guanidine hydrochloride, 2,4-dinitrophenylhydrazine (DNPH), ascorbic acid (AA), and trichloroacetic acid (TCA) were purchased from Coolaber Technology Co., Ltd. (Beijing, China). All other reagents were of analytical grade.

### 2.2. Preparation of the Purple Onion Pulp

Pre-cooled peeled onions were cut into pieces (1 cm × 1 cm) using a stainless-steel knife. Pre-cooled distilled water and onion pieces (ratio of water to onion pieces = 1:10) were then placed in a fruit and vegetable pulper to make onion puree (blade rotation speed 500 g/min, 3 min). The onion puree was stored at 4 °C for later use.

### 2.3. Preparation of Beef Patties

BPs were made according to the method of Abdelhakam et al. [24]. Fat and connective tissue were removed from beef loin and then the beef loin was immediately ground by a meat grinder (JR-32L, 6 mm holes, Zhucheng Huagang Machinery Co., Ltd., Zhucheng, China). The recipe for BPs contained the following ingredients: 1080 g beef, ice water (10%, *w*/*w*), salt (1.5%, *w*/*w*), and different proportions of OP (0%, 2.5%, 5.0%, and 10.0%, *w*/*w*), all percentages were calculated on the weight of beef [24]. BPs with 0.05% ascorbic acid (AA) were used as the positive control. Due to the large number of testing indicators, each group was set with 12 replicates to meet the sample requirements at different time points. All beef patty mixtures were first processed in a meat mincer. Subsequently, they were shaped into standard patties using a manual molding tool, with each patty weighing 80 g, with a diameter of 10 cm and a thickness of 1 cm. These patties were then sealed using a vacuum packaging machine (Model: DS3600, manufactured by Shenzhen Dingsheng Electric Appliance Co., Ltd., Guangdong, China) and stored under refrigeration at 4 °C. Various indicators of the BPs were tested and analyzed on days 0, 3, 6, 9, and 12. The basic chemical composition of the BPs is shown in Table 1.

### 2.4. pH Value Determination

The measurement of pH followed the method of Biswas et al. [25]. First, 10 g of the sample (*n* = 3) were ground with a mortar in 50 mL of distilled water. Afterwards, the pH value was measured with a digital pH meter (Five Easy Plus, METTLER TOLEDO instrument (Shanghai) Co., Ltd., Shanghai, China).

### 2.5. Color Determination

After the vacuum bags were opened, the beef patty samples were allowed to stand for 20 min, and then the meat color parameters (L*, a*, b*) were determined [15]. Three locations on each sample were measured, and the average value was taken. The colorimeter was calibrated using a standard white ceramic tile (L* = 101.28 ± 0.28, a* = 2.52 ± 0.12, b* = 3.21 ± 0.16). The total color difference (ΔE*) was calculated according to the method described by Bellucci et al. [26].

(1) $${∆E}^{*}=\sqrt{{\left(L_{3-12}^{*}-L_{0}^{*}\right)}^{2}+(a_{3-12}^{*}-a_{0}^{*}{)}^{2}+(b_{3-12}^{*}-b_{0}^{*}{)}^{2}}$$

where $L_{0}^{*}$, $a_{0}^{*}$, and $b_{0}^{*}$ are samples L*, a*, and b* on day 0; and $L_{3-12}^{*}$, $a_{3-12}^{*}$, and $b_{3-12}^{*}$ are the L*, a*, and b* values of the samples on days 3–12.

### 2.6. Cooking Loss Determination

Cooking loss of BPs was determined according to the method described by Bellucci et al. [26]. Samples (approximately 10 g of ground beef) packed in heat-sealed bags were heated in an 80 °C water bath until the core temperature reached 70 °C. Subsequently, the samples were cooled to room temperature (RT) and weighed. Cooking loss percentage was calculated from the weight difference between the raw and cooked samples, using the following formula:

(2) $$cooking\;loss\left(\%\right)=\frac{m_{1}-m_{2}}{m_{1}}\times 100$$

where m_1_ is mass of the raw samples and g; m_2_ is Mass of the cooked sample, g.

### 2.7. Texture Characteristics Determination

Texture profile analysis (TPA) of the BPs was performed according to the method described by Bahmanyar et al. [27], with slight modifications. Samples (length × width × height = 2 cm × 2 cm × 1 cm) were placed on the platform of a food texture analyzer (CT3-1500, Brookffeld Engineering Laboratories, Inc., Middleboro, MA, USA) to determine hardness (g), cohesiveness, springiness (mm), gumminess (g), and chewiness (g × mm). Gumminess was calculated as the ratio A2/A1, where A1 represents the total energy required for the first compression and A2 represents the total energy required for the second compression. Prior to analysis, the following instrumental parameters were established: probe type: 38.1 mm diameter cylindrical acrylic probe; pretest speed: 2.0 mm/s; test speed: 2.0 mm/s; and strain level: 50%.

### 2.8. Metmyoglobin (MetMb) Content Determination

The MetMb content in BPs was determined according to the methods described by Romero et al. [16] and Cui et al. [28]. A 3 g sample was homogenized with 30 mL of 0.04 mol/L phosphate-buffered solution (pH 6.8, 4 °C). The homogenate was then refrigerated at 4 °C for 1 h (incubation). Following incubation, the sample was centrifuged in a high-speed refrigerated centrifuge at 8000× *g* for 10 min at 4 °C. The resulting supernatant was filtered through Whatman No. 1 filter paper. The absorbance (A) of the filtrate was measured at 525 nm, 572 nm, and 700 nm using a UV-Vis spectrophotometer. The MetMb content was calculated using the following formula:

(3) $$MetMb\left(\%\right)=(1.395-\frac{{Abs}_{572}-{Abs}_{700}}{{Abs}_{525}-A_{700}})\times 100$$

### 2.9. Thiobarbitururic Acid Reactive Substances (TBARSs) Value

The TBARSs value was determined according to the method of Witte et al. [29], with slight modifications. A 10 g sample was homogenized in 25 mL of ice-cold 20% (*w*/*v*) trichloroacetic acid (TCA) solution using a mortar and pestle. The mortar was then rinsed with 25 mL of ice-cold distilled water and the rinse was combined with the homogenate in a 100 mL beaker. The mixture was filtered through filter paper. To 3 mL of the filtrate in a centrifuge tube, 3 mL of 5 mmol/L 2-thiobarbituric acid (TBA) reagent was added. The tube was incubated in a 95 °C water bath for 30 min. The reaction was terminated immediately by cooling in cold water. A reagent blank was prepared using 3 mL of 10% (*w*/*v*) TCA instead of sample filtrate. The absorbance of the sample was measured at 532 nm using a UV-Vis spectrophotometer. The TBARSs value was expressed as mg malondialdehyde (MDA) per kg sample (mg MDA/kg), calculated by multiplying the absorbance by a factor of 5.2.

### 2.10. Protein Carbonyl Content

The protein carbonyl content of BPs was measured as described by Vuorela [30]. A 1 g sample was mixed with 10 mL of ice-cold 0.15 mol/L potassium chloride (KCl) and homogenized using a mortar and pestle. Two 0.2 mL aliquots of the homogenate were separately treated with 1 mL of ice-cold 10% (*w*/*v*) TCA. The mixtures were then centrifuged at 5000× *g* for 5 min. The pellet from one aliquot was resuspended in 1 mL of ice-cold 2 mol/L hydrochloric acid (HCl) for protein quantification. The pellet from the other aliquot was resuspended in 1 mL of 0.2% (*w*/*v*) 2,4-dinitrophenylhydrazine (DNPH) in 2 mol/L HCl (ice-cold) for carbonyl quantification. After incubation at room temperature (RT) for 1 h, 1 mL of 10% (*w*/*v*) TCA was added to each tube, followed by centrifugation at 5000× *g* for 5 min. The resulting pellets were washed three times with 1 mL of an ethanol/ethyl acetate mixture (1:1, *v*/*v*) to remove unreacted DNPH. The final pellets were dissolved in 1.5 mL of 6 mol/L guanidine hydrochloride solution. After dissolution, the solutions were centrifuged for 2 min at 5000× *g* to clarify. Carbonyl content was determined spectrophotometrically by measuring absorbance at 370 nm, and protein content was determined by measuring absorbance at 280 nm.

### 2.11. Total Volatile Basic Nitrogen (TVB-N) Value

The TVB-N value was determined according to the method of Wang et al. [7]. A 10 g quantity of the sample was minced and mixed with 100 mL of distilled water. The mixture was extracted at room temperature for 30 min and then filtered. The filtrate was subjected to distillation using a semi-micro Kjeldahl distillation apparatus. Timing began when the first drop of condensate fell. The distillate was collected in a flask containing 10 mL of 2% (*w*/*v*) boric acid solution and 5–6 drops of a mixed indicator (prepared by combining 0.2% (*w*/*v*) methyl red solution and 0.1% (*w*/*v*) methylene blue solution in equal volumes). After exactly 5 min, the delivery tube was removed from the boric acid solution, and the distillate was immediately titrated with 0.01 mol/L hydrochloric acid (HCl) until the endpoint color changed to violet-blue (bluish-purple). A reagent blank was analyzed simultaneously. The TVB-N value was expressed as milligrams of volatile basic nitrogen per 100 g of beef (mg N/100 g).

(4) $$X(mg/100g)=\frac{(V_{1}-V_{2})\times M\times 14}{m\times 5/100}$$

where V_1_—Volume of HCl standard solution consumed in sample solution, mL;

V_2_—The volume of HCl standard solution consumed by the blank reagent, mL;

M—Molar concentration of hydrochloric acid standard solution, mol/L;

14—The amount of milligrams of equivalent nitrogen in 1 mL of 1 mol/L HCl standard solution;

m—Sample mass, g.

### 2.12. Total Aerobic Mesophilic Bacteria (TAMB) Determination

The *TAMB* in BPs during storage was determined using the pour plate method [7]. A 10 g quantity of the sample was homogenized in 90 mL of 0.85% sterile physiological saline for 30 min. Serial ten-fold dilutions were prepared using sterile physiological saline. One milliliter of the appropriate dilution was poured into a sterile Petri dish and mixed with approximately 15–20 mL of sterile plate count agar (PCA). After solidification, plates were incubated at 37 °C for 48 h. Following incubation, colonies were counted, and results were expressed as log_10_ colony-forming units per gram (log_10_ CFU/g).

### 2.13. Sensory Evaluation Analysis

Sensory evaluation of cooked BPs was conducted using descriptive analysis combined with a nine-point hedonic scale test within a protocol [7] approved by the Ethics Committee of Qiqihar University (Approval code: 20250312). A panel of thirty untrained assessors (10 males, 20 females) participated in the study. Each assessor evaluated raw patties from both the control group and AA-treated groups using the nine-point hedonic scale (where 1 = dislike extremely, 9 = like extremely). Attributes evaluated included color, odor, juiciness, hardness, and overall acceptability. All evaluations were performed in individual sensory booths. Samples, labeled with random three-digit codes, were presented to each assessor on white plates.

### 2.14. Statistical Analysis

For every experimental group, data were collected simultaneously under no fewer than three distinct conditions. To analyze the data statistically, one-way analysis of variance (ANOVA) was employed. In this analysis, a significance threshold of *p* < 0.05 was used to distinguish between statistically significant differences.

## 3. Results and Discussion

### 3.1. pH Value

Post-mortem, the anaerobic glycolysis of glycogen in beef produces a large amount of lactic acid and ATP-enzyme-catalyzed phosphorylation, both of which lead to a continuous decrease in the pH of the beef [7]. However, as the storage period extends, due to the influence of endogenous enzymes and microorganisms, the proteins and fats in the meat were broken down into small-molecule deaminated substances NH_3_ and amine compounds, causing the pH of the meat to gradually increase [30]. Table 2 depicts the pH changes in BPs during refrigeration. As the storage time extended, the pH in all groups increased by varying degrees, with addition of OP significantly reducing the pH of the BPs. The initial pH of the BPs was measured at between 5.35 and 5.46. After refrigeration for 3 days, the pH value of the control group (5.50) was significantly higher than that of the other treatment groups. After 12 days of refrigeration, the lowest pH was measured in the AA treatment group at 5.48, followed by 10.0% OP at 5.54 and 5.0% OP at 5.65. These results suggested that OP restrained the increase in pH of BPs during refrigeration. During refrigeration, the pH showed an upward trend, which is consistent with the report by researchers [24], attributed to the accumulation of alkaline substances produced by the microbial degradation of proteins [31]. The bioactive substances rich in OP (quercetin and kaempferol) effectively inhibited the proliferation of spoilage bacteria in the samples. Among twenty-eight vegetables and nine fruits, onions had the highest content of quercetin [31]. Moreover, quercetin and kaempferol have inhibitory effects on food spoilage bacteria, such as *Staphylococcus aureus*, *Bacillus cereus*, *Listeria monocytogenes* and *Micrococcus luteus* [32].

### 3.2. Color

The color of beef serves as a critical visual attribute for assessing its freshness. It is not only the primary criterion consumers use to evaluate shelf life and acceptability but also a key factor influencing their purchasing decisions [7]. Changes in beef color were influenced by the formation of MetMb and bacterial contamination [9]. Moreover, beef color is also dependent on factors such as pH, water-holding capacity, low oxygen level, lipid oxidative processes, and other parameters including feeding, genetics, and animal breed [14]. The changes in color parameters L*, a*, and b* of BPs during refrigeration are shown in Table 3. Neither refrigeration time nor addition level of OP had a significant influence on the L* value of BP, which is consistent with the study by Demir et al. [33]. As the storage time extended, the a* value gradually decreased, and the addition of OP decreased the a* value of the BP. This might be because the onion puree itself exhibits a pale purple color, which leads to a decrease in the a* value of the BP. On the 12th day of refrigeration, a* values across the treatment groups exhibited no significant differences. The decrease in a* value indicated discoloration of beef, that was, the oxidation of OxyMb (bright red) to MetMb (brown) [16]. The formation of MetMb was due to the oxidation of the heme group in Mb caused by free radicals produced by lipid oxidation, which in turn induces the browning of beef color [15]. This was consistent with the trend of metmyoglobin (MetMb) content in BP. During refrigeration, the increase in MetMb weakened the value of a* in the BP. Refrigeration time had no significant influence on the b* of BP, but the addition of OP and AA significantly reduced the b* value during storage. Furthermore, during the refrigeration process, the ΔE value of BPs gradually increased, with the smaller ∆E value of the 10.0% OP treatment group indicating that Mb oxidation was inhibited, attributed to the presence of polyphenols in onion pulp, enhancing the antioxidant and antimicrobial capabilities of the BPs.

### 3.3. Cooking Loss

**Figure 1:**
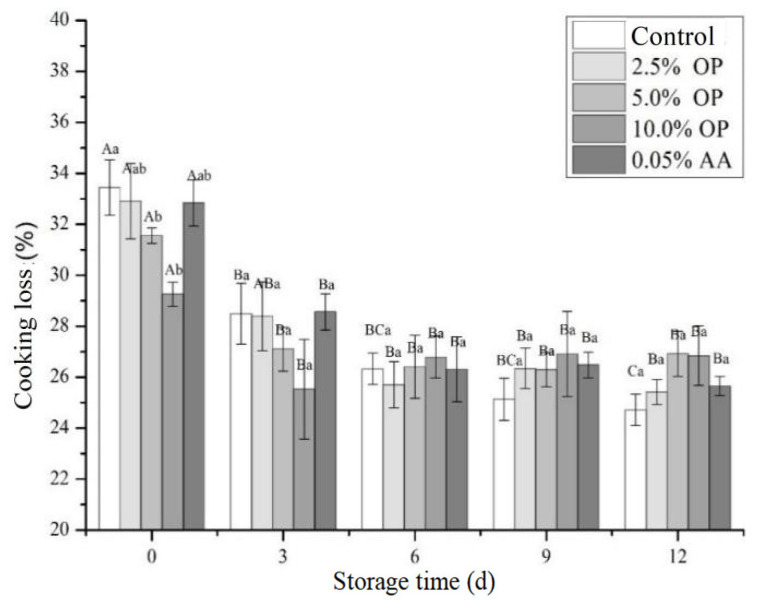
Influence of OP on cooking loss of beef patties during refrigeration. Different letters (A–C; a–b) indicate significant differences (*p* < 0.05) between means across days or across treatments, respectively. Means sharing the same letter do not differ significantly.

Cooking loss serves as an indicator of the water-holding capacity (WHC) of fresh meat, primarily measuring the loss incurred during the cooking process [26]. The degree of shrinkage and weight reduction occurring during the thermal processing of meat directly impacts its juiciness and texture. This shrinkage and concomitant weight loss involve the loss of not only water but also fat and soluble proteins. The spoilage of meat and meat products, as well as the water-binding capacity of exogenous additives, can alter the WHC of meat products, consequently affecting tenderness and product yield [26]. The effect of OP on cooking loss of BPs is shown in Figure 1. During the refrigerated storage period, no significant differences in cooking loss were found among various treatment groups. In the initial storage phase, the cooking process causes the denaturation of myofibrillar proteins and the shrinkage of collagen within the beef, leading to a reduction in its water-binding capacity and the consequent loss of substantial amounts of free water. On day 0, patties with 5.0% and 10.0% OP exhibited significantly lower cooking losses (31.56% and 29.26%, respectively) compared to the blank control group (33.44%). Although cooking loss decreased slightly after day 3, neither storage time nor additive treatment (OP or AA) had a significant effect. Throughout storage, the cold shortening induced by low temperature (4 °C) led to rigor mortis, decreased WHC, exudation of free water, and a consequent reduction in beef moisture content. During cooking, only the free water was expelled and leaked out through the widened spaces between myofibrils, along with some sarcoplasmic proteins, dissolved collagen, and fat [26]. In the initial storage period, the high content of carbohydrates in OP, particularly soluble fructans and insoluble plant fibers, likely contributed to moisture retention within the cooked meat [34]. As free water was released, the fructans within the patties decreased, and the water retained within the plant fibers was also lost due to thermal effects. This resulted in a slight increase in cooking loss for the OP-treated groups by storage day 12. Compared to the control and OP-treated groups, addition of AA had no significant influence on the cooking loss of the BPs. Increasing the addition level of common mushroom significantly decreased the cooking loss of BPs [15]. These results offer valuable guidance for the food industry in formulating strategies to improve meat product WHC. Water loss not only reduces product yield but also causes exudate accumulation within the packaging, damaging the color and texture of the meat, ultimately impacting the marketability and consumer acceptance of the meat products [15].

### 3.4. Texture Characteristics

The texture characteristics of beef were closely related to water and lipid content, and presence of water and fat in beef is beneficial to maintain the stability of beef texture [7]. Oxidation of proteins and lipids could result in the destruction of the cross-linked network structure, causing the loss of lipids and water in beef, thereby affecting the textural properties of BPs [34].

The impact of OP addition on the textural characteristics of BPs are displayed in Table 4. Addition of OP reduced the hardness, chewiness, and adhesiveness of the BP. The addition of 10.0% OP decreased the compressive strength (hardness) of the patties, but had no significant effect on the structure after the first compression (cohesion). The decrease in hardness may be associated with the relatively high water content in the BPs with 10% OP added (initial moisture content of beef was 74.42%, while water content of BPs with 10% onion pulp added was 77.68%). Meanwhile, this finding was consistent with the data on the cooking loss of the BPs. Furthermore, as the storage time extended, the elasticity, adhesiveness, and chewiness of the BPs exhibited an increasing and then decreasing trend. The structural characteristics of meat products have been closely associated with the properties of myofibrillar proteins [15]. Myofibrillar proteins were influenced by proteolytic enzymes in onions, resulting in increased tenderness of the samples [33]. Both red fruit and cactus fruit decreased the hardness of BPs, while cohesion was not affected by the berries [16]. Chewiness was determined by the combined product of hardness, cohesion, and elasticity. Among the three parameters, the addition of onion pulp had the greatest impact on hardness. Additionally, BPs with added onions had lower chew values in the final stage of refrigeration. Therefore, BPs made with onions were softer, requiring less energy to chew.

### 3.5. Metmyoglobin (MetMb) Content

**Figure 2:**
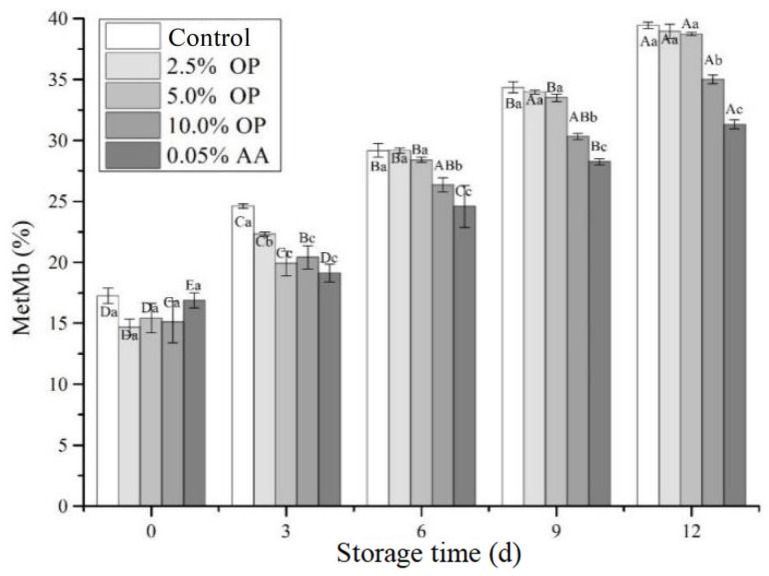
Effects of OP on MetMb content of BPs during refrigerated storage. Different letters (A–E; a–c) indicate significant differences (*p* < 0.05) between means across days or across treatments, respectively. Means sharing the same letter do not differ significantly.

Post-mortem, the beef myoglobin was exposed to air, prompting oxygen to combine with myoglobin to form oxymyoglobin (bright red). With the prolongation of refrigeration time, oxymyoglobin (OxyMb) is gradually oxidized to produce MetMb, and the color becomes brown, thus affecting the color of fresh meat. Therefore, the percentage of MetMb could be used to evaluate quality attributes of beef color [25]. In this study, we assessed the impact of various treatments on the percentage of MetMb in raw BPs and compared them with the control group stored for 12 days under refrigeration. The results are displayed in Figure 2. As the refrigeration time was extended, the MetMb content in all samples significantly increased. OP could slow down the oxidation rate of OxyMb to MetMb. At the beginning of refrigeration, MetMb content showed no significant difference between the control and experimental groups, ranging from 14.67% to 17.24%. By day 12, the MetMb content in the 10.0% OP group (35.01%) was lower than in the control (39.44%) yet higher than in the AA group (31.32%). This was due to the presence of a large amount of plant polyphenols and antioxidant active substances in the patties treated with OP, which reduced myoglobin oxidation and microbial growth. This was consistent with the trend of total aerobic mesophilic bacteria (TAMB) in BPs. On the other hand, the increase in MetMb content during the storage period originated from primary lipid oxidation products, such as hydroperoxides and other reactive species, which could oxidize the ferrous ions in OxyMb to the ferric form present in MetMb [22]. Noni puree has been shown to reduce the oxidation of Mb in BPs [14]. The addition of Kakadu plum (*Terminalia ferdinandiana*) and Opuntia ficus-indica fruits to BPs has been shown to delay the decrease in redness value of BPs during storage [9,16].

### 3.6. TBARSs Value

During food processing and storage, the problem of lipid oxidation is particularly prominent. In addition to impairing the sensory quality of food, this process can also lead to the production of harmful substances. [17]. Lipid oxidation consists of a sequence of reactions between unsaturated fatty acids and molecular oxygen, generating unstable primary products which can break down further into secondary ones like malondialdehyde (MDA) [7]. Polyphenols can react with both primary and secondary products of lipid oxidation, blocking or reducing the formation of harmful oxidation products, such as MDA and other volatile aldehyde compounds [35]. The TBARSs value, which quantifies aldehydes reacting with thiobarbituric acid, is associated with the accumulation of secondary products and is extensively utilized to indicate lipid oxidation [6].

**Figure 3:**
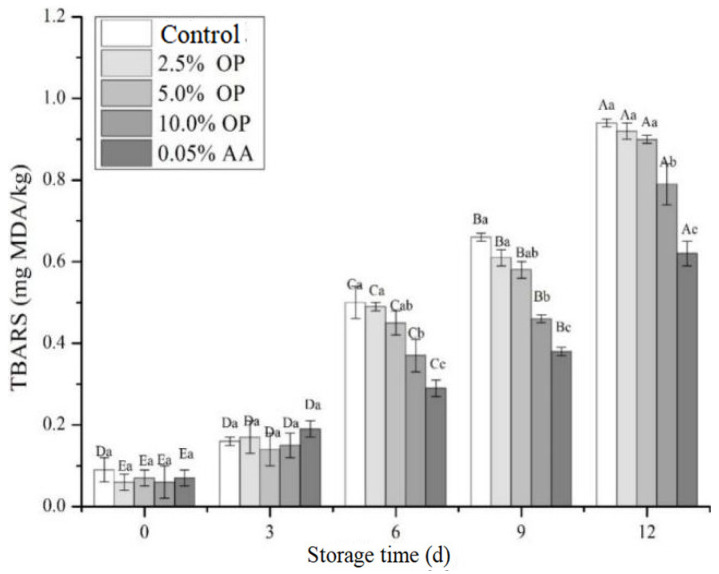
Effect of OP on TBARSs values of beef patties during refrigeration. Different letters (A–E; a–c) indicate significant differences (*p* < 0.05) between means across days or across treatments, respectively. Means sharing the same letter do not differ significantly.

The effect of adding OP on the MDA content produced by lipid oxidation in BPs is displayed in Figure 3. During refrigeration, MDA content in BPs showed an increasing tendency. From day 0 to day 3, there was no significant difference in MDA content among the BPs of different treatment groups. At the end of refrigeration, compared to the control, TBARSs values of the 2.5%, 5.0%, 10.0% OP, and AA treatment groups decreased by 2.13%, 4.26%, 15.96%, and 34.04%, respectively. Notably, the TBARSs values of the BPs treated with 2.5% and 5.0% OP exhibited no significant difference compared to the control (*p* > 0.05), while the addition of 10.0% OP significantly inhibited the formation of MDA. These data indicate that a higher addition of onion pulp can inhibit the lipid oxidation process in BPs during refrigeration. It is well known that natural antioxidants disrupt the free radical oxidation chain by providing hydrogen electrons from phenolic hydroxyl groups, thereby slowing down the lipid oxidation process [22]. In this study, the added onion pulp contained a high level of quercetin, which has antioxidant activity [34]. The quercetin level in chicken rolls with 50% onion juice was 5.68 mg/kg, effectively inhibiting the lipid oxidation of chicken rolls, while the initial concentration of quercetin in chicken rolls with 25% onion juice was too low to have any control effect on the subsequent development of lipid oxidation during refrigeration [36]. In addition, both Noni puree and red peppers have shown inhibitory effects on lipid oxidation in BPs, effectively preventing the deterioration of the flavor and texture of the patties [14,15].

### 3.7. Carbonyl Content

**Figure 4:**
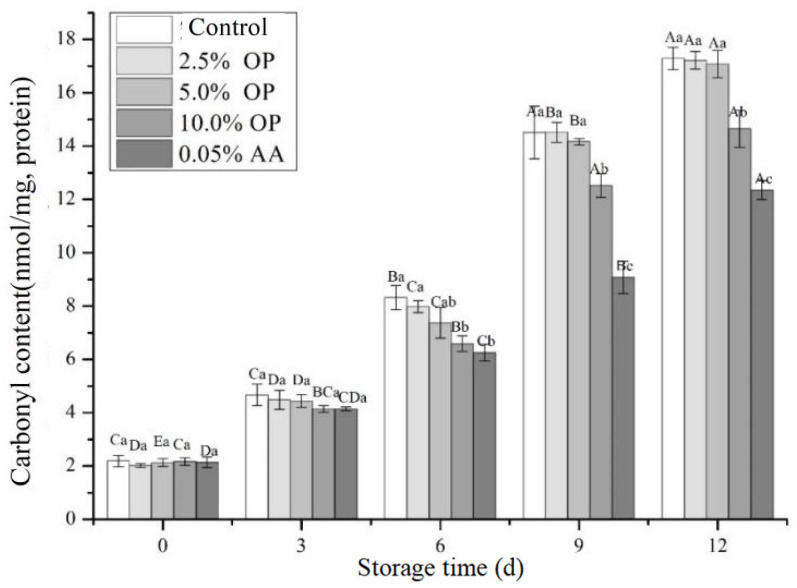
Effect of OP on carbonyl contents of beef patties during refrigeration. Different letters (A–D; a–c) indicate significant differences (*p* < 0.05) between means across days or across treatments, respectively. Means sharing the same letter do not differ significantly.

Protein carbonylation, a process predominantly induced by the reaction of amino acid residues with free radicals, reactive oxygen species (ROS), and aldehydes emanating from lipid oxidation, leads to the deterioration of meat quality through peptide bond scission, amino acid side-chain decomposition, and protein aggregation [34]. The influence of OP addition on protein carbonyl content in BPs was exhibited in Figure 4.

An extension in refrigeration duration led to a significant rise in carbonyl content within the BPs. However, on the third day of refrigeration, no significant differences in carbonyl content were observed among the various treatments (*p* > 0.05). Among samples stored for 6–12 days, the carbonyl content in both the OP and ascorbic acid (AA) treatment groups exhibited a variable decrease compared to the control group across all storage days. Notably, on the 12th day of refrigeration, the carbonyl content of patties supplemented with 10.0% OP was significantly reduced to 14.65 nmol/mg compared to the control. These results indicate that onion pulp, as a significant source of natural antioxidant additives, can effectively mitigate protein carbonyl formation, thereby reducing protein oxidation. This phenomenon might be attributed to the antioxidant properties of plant polyphenols, as these compounds can scavenge free radicals and ROS, in turn reducing protein oxidation and the subsequent generation of carbonyl groups [7]. Additionally, plant polyphenols may indirectly inhibit the process of protein carbonylation by suppressing the activity of enzymes associated with protein oxidation [5]. The addition of clove extract has been indicated to significantly suppress formation of protein carbonyls in BPs, highlighting the necessity of employing substantial doses of plant-derived antioxidant components to effectively curb protein oxidation [36]. Furthermore, mulberry polyphenols could restrain the oxidation of myofibrillar proteins in beef, consequently reducing carbonyl content during the oxidation process [37].

### 3.8. TVB-N Value

**Figure 5:**
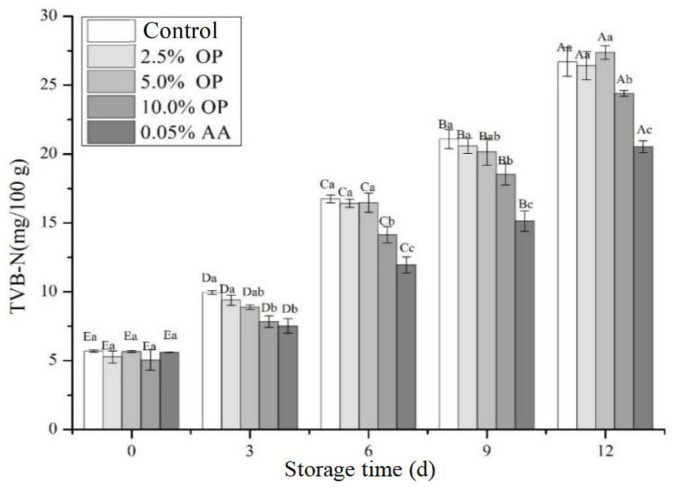
Effect of OP on TVB-N values of beef patties during refrigeration. Different letters (A–E; a–c) indicate significant differences (*p* < 0.05) between means across days or across treatments, respectively. Means sharing the same letter do not differ significantly.

**Figure 6:**
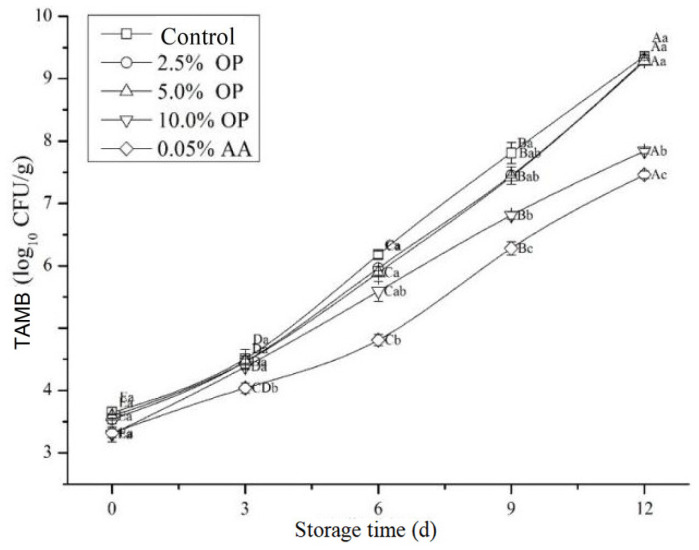
Effect of OP on TAMB of BPs during refrigeration. Different letters (A–E; a–c) indicate significant differences (*p* < 0.05) between means across days or across treatments, respectively. Means sharing the same letter do not differ significantly.

Endogenous enzymes and spoilage bacteria present in meat have the ability to break down proteins and other nitrogen-containing compounds into ammonia, trimethylamine, and dimethylamine, with these reactions leading to the formation of total volatile basic nitrogen (TVB-N) [38]. For this reason, TVB-N is regarded as one of the key indicators for assessing the quality of fresh meat. Notably, TVB-N values exhibit a strong correlation with the number of spoilage bacteria [39]. The higher the TVB-N value of the meat, the more severe the microbial contamination. The Chinese national standard stipulates that fresh meat should have a TVB-N value of ≤20 mg/100 g [7]. Figure 5 illustrates the variations in TVB-N content of BPs across different treatment groups over the storage period. The TVB-N values of BPs rose with the prolongation of refrigeration time. On the sixth day, the BPs from the 10.0% OP treatment group and the AA treatment group had significantly lower TVB-N values than the control (*p* < 0.05), and were still below the first-grade fresh meat standard (TVB-N ≤ 15 mg/100 g). Nevertheless, no significant difference in TVB-N was found between the BPs of the 2.5% OP treatment group, the 5.0% OP treatment group, and that of the control group—all of which were above the first-grade fresh meat standard (TVB-N ≤ 15 mg/100 g). By the ninth day of storage, the TVB-N level of the control group’s BPs had reached 21.09 mg/100 g, which exceeded the threshold value for second-grade fresh meat (20 mg/100 g). However, the TVB-N values of the 10.0% OP treatment group and the AA treatment group were 18.53 mg/100 g and 15.12 mg/100 g, respectively, both below 20 mg/100 g, remaining within the TVB-N value range for fresh meat. On the 12th day of storage, only the 0.05% AA treatment group’s TVB-N value remained below the threshold for second-grade fresh meat (20 mg/100 g), while the control group and OP treatment groups’ TVB-N values had both exceeded the threshold for second-grade fresh meat (20 mg/100 g). Moreover, the TVB-N values of BPs rose during refrigeration, which was consistent with changes in the total aerobic mesophilic bacteria (TAMB) (Figure 6). In conclusion, the addition of 10.0% OP to the BPs effectively reduced the TVB-N values during storage. This was mainly because onions contain a variety of antibacterial components, such as phenolic acids, flavonoids, and sulfur-containing compounds.

### 3.9. Total Aerobic Mesophilic Bacteria (TAMB)

TAMB reflects the degree of microbial contamination of food during production, processing, transportation, storage, and other processes [40]. Microbial contamination, an important reason for the spoilage and deterioration of meat products, accelerates the oxidative breakdown of proteins, leading to the loss of nutritional value and, ultimately, the reduction in commercial value [41,42]. Studies have shown that onions contain a variety of antibacterial components, such as phenolic compounds (quercetin) and organic sulfides (diallyl disulfide), etc. [32,43].

The change in the TAMB in BPs produced by mixing OP during refrigeration is shown in Figure 6. OP would effectively suppress the growth of TAMB of BPs during refrigeration. During refrigeration, the TAMB of all treatments increased significantly (*p* < 0.05). On the ninth day of refrigeration, the TAMB of control group exceeded 7 log10 CFU/g, which led to the spoilage of BPs. The TAMB of the meat patties treated with 10.0% OP and AA exceeded the upper limit of meat spoilage only on the 12th day. Compared with the control, the TAMB of the 10.0% OP and AA treatment groups decreased by 1.52 log10 CFU/g and 1.90 log10 CFU/g, respectively. Santas et al. analyzed the antibacterial activity of various onion components and pointed out that onion extracts have significant antibacterial activity against certain pathogens, including *Staphylococcus aureus*, *Bacillus cereus*, *Listeria monocytogenes*, and *Micrococcus luteus* [34]. Studies have demonstrated that onion extract significantly suppresses *Escherichia coli*, yeast, and mold in BPs under optimal conditions [44]. However, the minimal antibacterial effect observed at lower concentrations, as with 2.5% onion or 0.5% garlic, was often a phenomenon resulting from the inadequate application amount [45].

### 3.10. Sensory Evaluation

For BPs, sensory evaluation is of paramount importance for ensuring quality control and securing consumer acceptance, and their sensory quality directly affects consumers’ purchasing decisions and eating experience [46]. The sensory evaluation of BPs mainly includes indicators such as color, odor, succulence, hardness, and overall acceptability [47].

**Figure 7:**
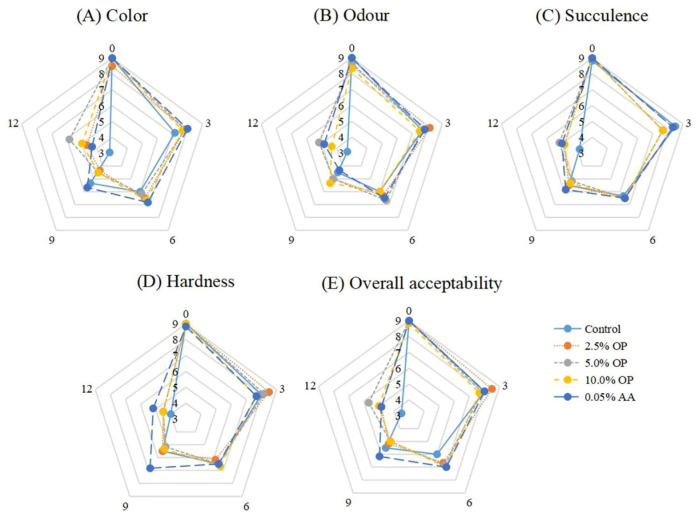
Effect of OP on sensory characteristics of BPs during refrigerated storage.

The sensory evaluation analysis of BPs prefabricated by mixing with purple onion pulp during refrigeration is shown in Figure 7. BPs prefabricated by mixing with OP have the highest sensory scores for color and odor. During storage, the quantity and odor intensity of sulfur-containing substances in onions increase, including 2-propen-1-thiol, 3,3-thiobis-1-propene, and methyl 2-propenyl disulfide, which overcome the odors produced by methanethiol, dimethyl disulfide, and dimethyl trisulfide and enhance the aroma of beef [44]. The higher color score was related to the inhibition of Mb oxidation by active ingredients such as quercetin and kaempferol in onion pulp, delaying the conversion of OxyMb to MetMb [28]. On the final day of storage, BPs with OP still maintained a succulence state, but there was no significant improvement in hardness (*p* > 0.05). The enhanced water-holding capacity of the muscle was due to sulfides and polyphenols from onions, which inhibited microbial growth and reduced the contraction and damage of structural proteins, thereby preventing water loss from the gel structure [48]. According to the overall acceptability score, onions can effectively reduce the impact of oxidation and microbial contamination on the sensory characteristics of meat products. Adding minced onions (2.5%) to BPs has no significant effect on the overall sensory acceptance of cooked meat [45]. BPs incorporating an addition of carrot pomace of no more than 3% did not differ significantly in aroma, taste, appearance, or overall acceptability. In contrast, BPs incorporating an addition of carrot pomace (4.2%) scored lower in terms of overall preference and firmness [49]. In addition, adding 5% raspberry pomace or blackberry pomace increased the cellulose content in BPs, resulting in a marginal increase in beef patty hardness [50]. Thus, it can be seen that the overall sensory characteristics of BPs were significantly related to the addition amount of OP.

## 4. Conclusions

OP is an effective, natural, plant-based additive that can enhance the oxidative stability and microbial safety of meat products, while improving their sensory quality. Notably, the treatment with 10.0% OP exhibits the most significant effect: it can remarkably inhibit the accumulation of lipid oxidation products (MDA), protein carbonyl compounds, and spoilage microorganisms growth, and provide a shelf-life extension of up to 9 days in BPs. Sensory evaluation further indicates that the addition of OP improves the color and flavor attributes without exerting adverse effects on the overall acceptability. This research suggested valuable practical insights into the application of plant-derived ingredients in enhancing the quality and prolonging the shelf life of meat. It offers support for the development of meat formulations with cleaner labels, so as to meet consumers’ growing demand for natural and functional food ingredients. However, this research has certain limitations, including focusing only on one vegetable additive and comparing it with a single synthetic antioxidant. In the future, further research is required to explore the synergistic effects between OP and other natural preservatives, evaluate the impacts under different storage conditions, and verify the sensory results through larger-scale consumer panels. Future studies should also aim to optimize the additive dosage and assess its economic feasibility in food industrial applications. Notwithstanding its contributions, this research was subject to several limitations. For instance, it only focuses on the comparison between a single vegetable and AA. In the future, it is imperative to explore the synergistic effects of onion puree with other natural food quality improvers on enhancing the color attributes and textural properties of BPs, and verify sensory effects through larger-scale consumer tests. Subsequent studies should also optimize the addition level of natural food additives and evaluate their economic feasibility in food industry applications.

## Figures and Tables

**Table 1:** Basic chemical composition content in BPs.

Content	Moisture (%)	Protein (%)	Fat (%)
Control	74.42 ± 1.22	19.25 ± 0.12	2.15 ± 0.13
2.5% OP	75.44 ± 0.73	18.26 ± 0.21	2.01 ± 0.14
5.0% OP	75.87 ± 0.58	17.86 ± 0.31	1.95 ± 0.17
10.0% OP	77.68 ± 0.37	17.15 ± 0.23	1.82 ± 0.11
0.05% AA	74.58 ± 0.58	19.34 ± 0.25	2.19 ± 0.16

**Table 2:** Effect of OP on pH value of BPs during refrigeration.

Storage Time (D)	Control	2.5% OP	5.0% OP	10.0% OP	0.05% AA
0	5.56 ^Da^ ± 0.01	5.43 ^Ca^ ± 0.01	5.44 ^Ca^ ± 0.02	5.45 ^Ba^ ± 0.03	5.35 ^Cb^ ± 0.04
3	5.50 ^Ca^ ± 0.01	5.44 ^Cb^ ± 0.01	5.44 ^Cb^ ± 0.01	5.45 ^Bb^ ± 0.01	5.42 ^Bc^ ± 0.01
6	5.53 ^Ca^ ± 0.01	5.47 ^Cb^ ± 0.03	5.48 ^Bb^ ± 0.01	5.45 ^Bc^ ± 0.02	5.41 ^Bc^ ± 0.01
9	5.62 ^Ba^ ± 0.04	5.55 ^Bab^ ± 0.03	5.50 ^Bb^ ± 0.01	5.46 ^Bb^ ± 0.01	5.33 ^Cb^ ± 0.03
12	5.73 ^Aa^ ± 0.02	5.72 ^Aa^ ± 0.02	5.65 ^Ab^ ± 0.01	5.54 ^Ac^ ± 0.01	5.48 ^Ad^ ± 0.01

Different letters (A–D for columns; a–d for rows) indicate significant differences (*p* < 0.05) between means within a column (across days) or row (across treatments), respectively.

**Table 3:** Influence of OP on color of BPs during refrigeration.

Color Parameters	Storage Time (D)	Control	2.5% OP	5.0% OP	10.0% OP	0.05% AA
L*	0	31.4 ^Aa^ ± 0.21	31.1 ^Aa^ ± 1.53	31.4 ^Aa^ ± 0.67	30.0 ^Aa^ ± 0.10	31.1 ^Aa^ ± 0.17
	3	31.6 ^Aa^ ± 0.39	31.2 ^Ab^ ± 0.06	30.8 ^Cb^ ± 0.29	31.3 ^Ab^ ± 0.44	31.2 ^Aa^ ± 0.16
	6	31.7 ^Aa^ ± 1.90	31.9 ^Aa^ ± 1.5	31.1 ^Ba^ ± 0.85	31.6 ^Aa^ ± 1.42	31.7 ^Aa^ ± 0.47
	9	32.0 ^Aa^ ± 1.28	31.8 ^Aa^ ± 2.43	30.9 ^Ca^ ± 0.64	31.9 ^Aa^ ± 0.65	31.3 ^Aa^ ± 0.23
	12	32.7 ^Aa^ ± 0.38	31.8 ^Aa^ ± 0.49	32.5 ^Aa^ ± 1.11	31.4 ^Aa^ ± 1.48	32.0 ^Aa^ ± 1.22
a*	0	12.4 ^Aa^ ± 1.49	10.6 ^Bab^ ± 0.70	9.3 ^Cb^ ± 0.19	9.0 ^Cb^ ± 0.21	11.7 ^Aa^ ± 0.14
	3	12.4 ^Aa^ ± 0.39	10.3 ^Ab^ ± 0.45	10.7 ^ABb^ ± 0.39	10.4 ^Ab^ ± 0.06	11.2 ^Aab^ ± 1.00
	6	12.1 ^Aa^ ± 0.53	11.1 ^Aa^ ± 2.35	11.3 ^Aa^ ± 1.28	10.0 ^Aa^ ± 0.59	11.5 ^Aa^ ± 2.25
	9	11.9 ^Aa^ ± 0.94	9.1 ^Ac^ ± 1.55	10.0 ^ABbc^ ± 0.11	9.2 ^BCc^ ± 0.56	11.2 ^Aab^ ± 0.76
	12	10.1 ^Aa^ ± 0.74	9.4 ^Aa^ ± 0.45	10.7 ^ABa^ ± 0.67	9.9 ^ABa^ ± 0.16	9.4 ^Aa^ ± 1.42
b*	0	7.6 ^Aa^ ± 0.81	7.1 ^Aa^ ± 0.98	6.9 ^Ba^ ± 0.21	6.6 ^Aa^ ± 0.18	6.6 ^Aa^ ± 0.73
	3	8.0 ^Aa^ ± 0.41	7.7 ^Aa^ ± 0.23	7.0 ^Ba^ ± 0.14	7.4 ^Aa^ ± 0.59	7.8 ^Aa^ ± 0.13
	6	8.2 ^Aa^ ± 0.57	8.0 ^Aa^ ± 0.23	7.9 ^Aa^ ± 0.77	7.4 ^Aa^ ± 0.50	7.7 ^Aa^ ± 0.38
	9	8.8 ^Aa^ ± 0.43	7.6 ^Ab^ ± 0.67	7.0 ^Bb^ ± 0.19	7.3 ^Ab^ ± 0.34	7.7 ^Ab^ ± 0.47
	12	9.2 ^Aa^ ± 0.64	7.9 ^Ab^ ± 0.30	8.1 ^Aab^ ± 0.47	7.2 ^Ab^ ± 0.79	7.9 ^Ab^ ± 0.66
ΔE	0–3	2.63 ^Ba^ ± 0.10	1.99 ^Ba^ ± 0.64	2.72 ^ABa^ ± 0.24	2.63 ^Aa^ ± 0.25	2.45 ^ABa^ ± 0.12
	0–6	3.35 ^ABa^ ± 0.72	4.11 ^Aa^ ± 1.01	3.39 ^ABa^ ± 0.35	2.60 ^Aa^ ± 0.22	2.52 ^ABa^ ± 0.50
	0–9	2.76 ^ABa^ ± 0.53	3.19 ^ABa^ ± 0.42	2.44 ^Ba^ ± 0.82	2.40 ^Aa^ ± 0.73	1.81 ^Ba^ ± 0.06
	0–12	4.94 ^Aa^ ± 0.92	2.86 ^ABb^ ± 0.75	4.24 ^Aab^ ± 0.61	2.38 ^Ab^ ± 0.57	3.94 ^Aab^ ± 0.94

Different letters (A–C for columns; a–c for rows) indicate significant differences (*p* < 0.05) between means within a column (across days) or row (across treatments), respectively.

**Table 4:** Influence of OP on texture characteristics of beef patties during refrigeration.

Properties	Storage Time (D)	Control	2.5% OP	5.0% OP	10.0% OP	0.05% AA
Hardness (N)	0	24.37 ^Ba^ ± 0.80	26.52 ^Aa^ ± 1.77	26.03 ^Aa^ ± 0.63	25.76 ^Aa^ ± 1.23	26.98 ^Ba^ ± 2.47
	3	33.72 ^Aa^ ± 3.50	29.09 ^Aab^ ± 1.95	25.17 ^Abc^ ± 1.01	23.43 ^Ac^ ± 1.06	33.44 ^Aa^ ± 4.67
	6	37.10 ^Aa^ ± 6.80	31.96 ^Aab^ ± 3.31	27.29 ^Ab^ ± 4.12	25.15 ^Ab^ ± 0.02	36.97 ^Aa^ ± 1.01
	9	36.88 ^Aa^ ± 2.39	31.57 ^Aab^ ± 1.17	28.71 ^Aab^ ± 3.06	25.29 ^Ab^ ± 2.23	34.55 ^Aab^ ± 0.35
	12	35.90 ^Aa^ ± 0.58	30.87 ^Aab^ ± 5.43	27.06 ^Aab^ ± 5.13	24.63 ^Ab^ ± 2.99	34.94 ^Aa^ ± 0.52
Cohesiveness	0	0.24 ^Aa^ ± 0.01	0.26 ^ABa^ ± 0.01	0.26 ^Aa^ ± 0.01	0.25 ^Aa^ ± 0.02	0.26 ^Aa^ ± 0.01
	3	0.26 ^Aa^ ± 0.01	0.26 ^ABa^ ± 0.00	0.27 ^Aa^ ± 0.01	0.26 ^Aa^ ± 0.02	0.27 ^Aa^ ± 0.01
	6	0.25 ^Ab^ ± 0.01	0.28 ^Aa^ ± 0.02	0.25 ^Ab^ ± 0.01	0.26 ^Ab^ ± 0.01	0.27 ^Aab^ ± 0.01
	9	0.25 ^Aa^ ± 0.02	0.25 ^Ba^ ± 0.01	0.26 ^Aa^ ± 0.02	0.25 ^Aa^ ± 0.01	0.26 ^Aa^ ± 0.02
	12	0.25 ^Aa^ ± 0.01	0.25 ^Ba^ ± 0.01	0.26 ^Aa^ ± 0.02	0.25 ^Aa^ ± 0.01	0.27 ^Aa^ ± 0.03
Springiness (mm)	0	0.97 ^Aa^ ± 0.14	1.00 ^Ca^ ± 0.06	0.94 ^Ba^ ± 0.04	1.13 ^ABa^ ± 0.01	1.03 ^Ba^ ± 0.23
	3	1.17 ^Aa^ ± 0.07	1.08 ^BCab^ ± 0.08	1.02 ^Bbc^ ± 0.06	0.94 ^Bc^ ± 0.04	1.16 ^Ba^ ± 0.05
	6	1.30 ^Aa^ ± 0.07	1.09 ^BCc^ ± 0.03	1.02 ^Bc^ ± 0.05	1.16 ^ABbc^ ± 0.015	1.48 ^Aa^ ± 0.16
	9	1.28 ^Aa^ ± 0.03	1.41 ^Aa^ ± 0.11	1.29 ^Aa^ ± 0.22	1.30 ^Aa^ ± 0.08	1.33 ^ABa^ ± 0.16
	12	1.21 ^Aa^ ± 0.10	1.23 ^ABa^ ± 0.19	1.32 ^Aa^ ± 0.15	1.12 ^ABa^ ± 0.27	1.11 ^Ba^ ± 0.13
Gumminess (g)	0	7.82 ^Aa^ ± 0.11	7.84 ^Aa^ ± 0.26	6.79 ^Ab^ ± 0.16	6.58 ^ABb^ ± 0.71	6.86 ^Cb^ ± 0.79
	3	8.70 ^Aa^ ± 0.99	7.59 ^Aab^ ± 0.60	6.80 ^Ab^ ± 0.14	6.14 ^Bb^ ± 0.63	8.94 ^ABa^ ± 1.28
	6	9.37 ^Aab^ ± 1.43	8.91 ^Aab^ ± 1.05	6.91 ^Ac^ ± 1.14	7.56 ^Abc^ ± 0.42	9.85 ^Aa^ ± 0.51
	9	9.85 ^Aa^ ± 0.85	9.46 ^Aa^ ± 2.06	7.32 ^Aa^ ± 0.61	6.22 ^Ba^ ± 0.50	8.25 ^ABa^ ± 1.25
	12	7.66 ^Aa^ ± 0.23	7.58 ^Aa^ ± 1.30	6.79 ^Aa^ ± 2.08	6.21 ^Ba^ ± 0.85	7.28 ^BCa^ ± 0.41
Chewiness (g·m)	0	7.65 ^Ba^ ± 0.11	7.83 ^Ba^ ± 0.26	6.39 ^Ba^ ± 0.16	7.45 ^ABb^ ± 0.71	7.10 ^Ca^ ± 0.79
	3	10.25 ^ABa^ ± 0.99	8.21 ^Bab^ ± 0.60	6.97 ^ABbc^ ± 0.14	5.77 ^Bc^ ± 0.63	10.22 ^BCa^ ± 0.28
	6	12.19 ^Aab^ ± 0.43	9.72 ^ABbc^ ± 1.05	7.09 ^ABc^ ± 1.14	8.80 ^ABc^ ± 0.42	14.58 ^Aa^ ± 0.51
	9	12.56 ^Aa^ ± 0.85	13.48 ^Aa^ ± 0.60	9.45 ^ABb^ ± 0.61	8.05 ^Ab^ ± 0.50	11.03 ^ABab^ ± 0.25
	12	9.28 ^ABa^ ± 0.23	9.49 ^ABa^ ± 0.30	9.89 ^Aa^ ± 0.18	6.88 ^ABb^ ± 0.85	8.08 ^Cab^ ± 0.41

Different letters (A–C; a–c) indicate significant differences (*p* < 0.05) between means across days or across treatments, respectively. Means sharing the same letter do not differ significantly.

## Data Availability

All data generated or analyzed during this study were included in this article.
